# Recent advances in the CRISPR genome editing tool set

**DOI:** 10.1038/s12276-019-0339-7

**Published:** 2019-11-05

**Authors:** Su Bin Moon, Do Yon Kim, Jeong-Heon Ko, Yong-Sam Kim

**Affiliations:** 1 Genome Editing Research Center, KRIBB, Daejeon, Republic of Korea; 2 KRIBB School of Bioscience, Korea University of Science and Technology (UST), Daejeon, Republic of Korea

**Keywords:** Genetic engineering, Genetic techniques

## Abstract

Genome editing took a dramatic turn with the development of the clustered regularly interspaced short palindromic repeats (CRISPR)-CRISPR-associated proteins (Cas) system. The CRISPR-Cas system is functionally divided into classes 1 and 2 according to the composition of the effector genes. Class 2 consists of a single effector nuclease, and routine practice of genome editing has been achieved by the development of the Class 2 CRISPR-Cas system, which includes the type II, V, and VI CRISPR-Cas systems. Types II and V can be used for DNA editing, while type VI is employed for RNA editing. CRISPR techniques induce both qualitative and quantitative alterations in gene expression via the double-stranded breakage (DSB) repair pathway, base editing, transposase-dependent DNA integration, and gene regulation using the CRISPR-dCas or type VI CRISPR system. Despite significant technical improvements, technical challenges should be further addressed, including insufficient indel and HDR efficiency, off-target activity, the large size of Cas, PAM restrictions, and immune responses. If sophisticatedly refined, CRISPR technology will harness the process of DNA rewriting, which has potential applications in therapeutics, diagnostics, and biotechnology.

## Genome editing: Re-writing DNA

Yong-Sam Kim and colleagues at Korea Research Institute of Bioscience and Biotechnology in Daejeon, South Korea, outline the latest developments in genome editing technologies and the limitations that remain. Although several approaches have been developed to make changes to an organism’s DNA at particular locations in the genome, the efficiency and simplicity of the CRISPR-Cas system, which plays a defense role in bacteria or archaea by chopping up the invaded DNA, has revolutionized the field. The authors review the different types of CRISPR system and explain how they induce quantitative or qualitative changes in gene expression in eukaryotic cells. Further improving the system’s DNA modification efficiency and the delivery of its components into cells, and finding ways to reduce off-target effects, will be required for CRISPR to realize its therapeutic potential.

## Introduction

Genome editing technology is a type of engineering by which intracellular DNA is modified in a sequence-specific manner. The modifications include insertions, deletions, integrations, and sequence substitutions. Studies on the repair mechanisms underlying DNA damage and the resulting structural changes in DNA have formed the basis of targeted genome editing^1^. In addition, site-specific genetic or epigenetic regulations became possible by combining sequence-identifiable programmable nucleases and regulatory proteins. Early developments in genome editing technology have been made by the sophisticated engineering of gene-targetable sequence identifiers. The concept of targeted genome editing was explored by the development of a meganuclease, which was initially created by the fusion of the catalytically active nuclease domain of FokI and engineered I-SceI with a sequence-targeting ability for 18 base pairs^2^. Zinc finger nuclease (ZFN) uses zinc finger modules, each of which recognizes a 3-nt DNA sequence. A pair of fusion proteins composed of an array of zinc finger modules and a FokI nuclease domain induce double-stranded breakages (DSBs) in a targeted site. Transcription activator-like effector nucleases (TALENs) employ a similar platform to that of ZFN except that ZF proteins are replaced by 14-24 TALENs, each of which specifically recognizes a 1-bp oligonucleotide through the different base specificity of the repeat variable diresidue (RVD)^3^. ZFNs and TALENs opened up a bona fide genome editing era, but there were several limitations to their routine use as genome editing tools: there is a limit to the targetable sequences for zinc fingers and, furthermore, a redundancy in ZF-DNA match. Although the open-source ‘Oligomerized Pool Engineering (OPEN)’ protocol has helped to create potent ZFN modules^4^, it requires a complex step to secure optimized modules. Purchasing optimized modules was also costly. TALENs have low off-target efficiency and flexibility for target selection compared to ZFNs, but it is time-consuming to construct TALE modules and the endonuclease domain^5^. Despite the technical developments to overcome these hurdles, there remained an overall low efficiency in the use of those techniques as a versatile genome editing platform.

Clustered regularly interspaced short palindromic repeats (CRISPR) is an acquired immune system in archaea and bacteria that involves CRISPR-associated nuclease (Cas) in the modification of invading nucleotides^6^. The process by which the immune system operates is divided into three stages: (i) adaptation, (ii) expression, and (iii) interference. Adaptation is the process of inserting foreign DNA fragments into the CRISPR locus of a host chromosome with the aid of Cas1 and Cas2^7^. The expression stage includes the production of guide RNA (gRNA) and maturation of transcribed pre-gRNA. In the interference step, the cleavage of invaded DNA is catalyzed by Cas protein bound to the maturated gRNA. The Cas-gRNA complex recognizes a protospacer adjacent motif (PAM) and then stays at a sequence-matched protospacer region. The structural modification of the Cas protein induced by this process activates nuclease activity and cleaves target DNA^8^. Cleavage by PAM recognition prevents attack against the bacterial CRISPR locus^9^. The RNA-guided CRISPR-Cas system includes recognition and cleavage as key elements of the genome editing tool. The process of CRISPR biology has been directly applied to DNA modifications in both prokaryotic and eukaryotic cells^10,11^. In addition to ease, simplicity, and flexibility in practical use, CRISPR technology guarantees high DNA modification efficiency in a wide range of target genes. Moreover, it offers a platform for multiplexed and upscaled genome editing.

It is known that most bacterial and archaeal cells deploy the CRISPR system in their chromosomes^12^. However, the system has been diversified during evolution, and the mode by which it works differs depending on the Cas gene and the production of gRNA, which offers a variety of options in genome editing. Therefore, it is necessary to understand a spectrum of CRISPR systems that are distributed in a wide range of prokaryotic cells and to employ each system for tailored genetic engineering. We will also introduce recent advances in the technological developments in the practice of CRISPR as well as challenges to the *status quo* that await further refinements.

### Classification of the CRISPR system

The CRISPR-Cas system is functionally divided into two classes according to the composition of the effector nuclease genes. The class 1 CRISPR system is characterized by multisubunits of effector nuclease complexes and includes the type I, III, and IV CRISPR systems. Although several CRISPR systems belonging to Class 1 were reported in terms of the intracellular process underlying the defense mechanisms^13^, routine applications of the class as a genome editing tool have been limited because of not only limited knowledge but also restrictions in the cloning of the system in a functional vector or in the use of a ribonucleoprotein protein (RNP) complex. Thus, we will focus on the class 2 system in this review, which offers an opportunity for a variety of genetic engineering and DNA modification strategies.

**Fig. 1:**
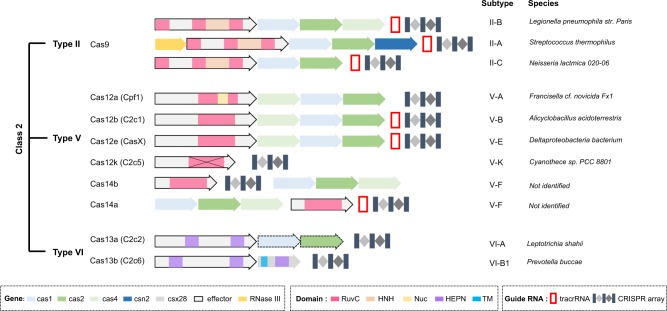
Classification of the CRISPR system and the operon organization for each subtype. Genes belong to any step composed of the chromosomal integration of invaded DNA (adaptation), the production of CRISPR RNA (expression), and the cleavage of exogenous DNA (interference). The class 2 CRISPR system is characterized as a single nuclease effector protein, and type II, V, and VI constitute the Class 2 system. Types II and V show DNA cleavage activity, while type VI uses ssRNA as a substrate. A systematic name is given for all effector nucleases with a legacy name in parentheses for several nucleases. The dashed arrows indicate that cas1 and cas2 genes are present in only some bacterial or archeal chromosomes. The X mark denoted in Cas12k refers to a catalytically inactive RuvC-like domain

**Fig. 2:**
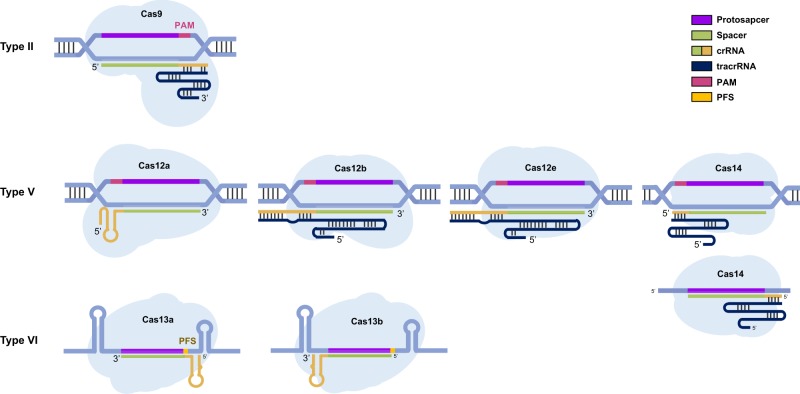
Validated genome editing tools in the Class 2 CRISPR system and the architecture in the cleavage site. Cas9 represents a type II system and is guided by crRNA and tracrRNA. The RNP form recognizes the compatible PAM sequence and opens the helical structure when sequence similarity between the crRNA and protospacer exists. Various subtypes have been validated in type V, including Cas12a, Cas12b, Cas12e, and Cas14, where each subtype shows a distinct architecture. Only crRNA is required for Cas12a, while the other subtypes require an additional tracrRNA. Cas14 shows both ssDNA and dsDNA cleavage activity. A PAM requirement exists for dsDNA cleavage. The best characterized RNA nuclease Cas13 belongs to Type VI. In recognition of the PFS sequence, the crRNA-Cas13 complex induces ssRNA cleavage activity. Upon target recognition, Cas13 is armored with collateral ssRNA activity

A class 2 CRISPR system consists of a single effector protein such as Cas9 and is subclassified into types II, V, and VI^14^ according to the factors necessary for pre-crRNA processing and the diversity of the domains constituting the effector protein. Because each type shows different specificity for nucleotide substrates and PAM, cleavage pattern, and other distinct features, a close look at each type of Class 2 CRISPR system would facilitate its deployment as a suitable tool for tailored and fine-tuned genome engineering (Table 1). The classification of the CRISPR system is provided with the operon organization with a focus on the class 2 system (Fig. 1). The subtypes that were validated for use as a genome editing tool were introduced in Fig. 2 in terms of the structural elucidations of Cas in the complex with guide RNAs and targeted DNA or RNA substrates.

**Table 1:** Properties of validated genome editing tools for the class 2 CRISPR system

Subtype	II/Cas9	V-A/Cas12a	V-B/Cas12b	VI-A/Cas13a	V-F/Cas14	V-E/Cas12e
characteristics						
Substrate	dsDNA	dsDNA	dsDNA	ssRNA	ssDNA dsDNA	dsDNA
Guide RNA components	crRNA tracrRNA	crRNA	crRNA tracrRNA	crRNA	crRNA tracrRNA	crRNA tracrRNA
PAM/PFS	3’-GG	5’-TT(T)	5’-TT(T)	3’-H^ a^	5’-TT(T)	5’-TT(T)
Effector protein size (kb)	2.9–4.9	3.6–3.9	3.4	4.1	1.2–2.1	2.95
Target sequence length (nt)	20–24	20–24	20–25	20	20–25	20
Cleavage pattern	Staggered 5’-overhang^ b^	Staggered 5’-overhang (collateral ssDNA cleavage)	Staggered 5’-overhang	gRNA-dependent RNA cut (collateral RNA cleavage)	gRNA-dependent ssDNA and dsDNA cuts (collateral ssDNA cleavage)	Staggered 5’-overhang

^a^A, T, or C

^b^A staggered 5’-overhang is produced following the postcleavage trimming activity of the RuvC domain

### Type II CRISPR system

The era of CRISPR technology has emerged from applications of the type II-A (SpCas9, Cas9 from *Streptococcus pyogenes*) subtype to genome editing in eukaryotic systems^15^. Type II-A is distinct from other Cas proteins, including type V and type VI, because it includes RNase III genes for the maturation of pre-crRNA. In complex with mature gRNA, Cas9 recognizes a G-rich PAM sequence and is directed to a target DNA that is complementary to the spacer sequence of crRNA. PAM recognition induces a structural alteration in Cas, resulting in unwinding of target DNAs to generate an R-loop formation between gRNA and target DNA^16^. The HNH and RuvC domains are involved in the cleavage of target and nontarget strands, respectively^17^. Previously, it was believed that SpCas9 creates blunt-end DSBs, but a recent publication revealed the formation of a staggered end with 5 ‘-overhangs due to the postcleavage trimming activity of the RuvC domain^18^. Type II-B / C requires tracrRNA for target recognition similar to Type II-A. Type II-B, FnCas9, recruits a complex of small CRISPR-Cas associated RNA (scaRNA) and tracrRNA for targeting and degrades mRNA^19^. Type II-C *Neisseria lactamica* Cas9 (NmCas9) has RNase III independent RNA processing^20^. Each Cas9 ortholog shows diversity in PAM sequence, size, spacer length, and other genome editing properties. In particular, *Staphylococcus aureus* Cas9 (SaCas9) and *Campylobacter jejuni* Cas9 (CjCas9) have a smaller size than SpCas9, which made packaging into AAV vectors attainable^21^. The split system using N- and C-SpCas9 fragments offers an alternative genome editing option in viral vector-based delivery systems^22^.

### Type V CRISPR system

The type V, Class 2 system, represented by Cas12a (formerly known as Cpf1), is subdivided into 10 known subtypes from A-I to U according to the similarity of the domain organization^23^. Cas12b was reported to have null activity at 37 °C^24^, but *Bacillus hisaishi* Cas12b (BhCas12b) and its engineered variant, BhCas12b v4, was suggested as a feasible genome editing tool in vivo^25^. Similar to the type II system, the type V nucleases constitute a bilobed architecture comprising recognition (REC) and nuclease (NUC) lobes. Unlike type II, however, they possess only the RuvC domain in the NUC lobe in which the HNH domain is depleted^26^. This architecture is identically observed in Cas12a, Cas12b (C2c1), Cas12e (CasX), and Cas14^27–30^. The composition of gRNA is different among subtypes. Whereas Cas12a requires only crRNA, an additional tracrRNA is necessary for Cas12b, Cas12e, and Cas14a^23^. Cas12a is also unique in that it possesses RNA editing activity and thus trims pre-crRNA to mature crRNA^31^. The type V system usually shows specificity toward T-rich PAM sequences located 3’-upstream of a protospacer^14,23^. Cas14 cleaves ssDNA without PAM specificity^30^, but a recent study identified dsDNA cleavage through the recognition of T-rich PAM (TTTR/TTAT) sequences^32^. Structural changes in the NUC lobe upon PAM recognition induce target DNA cleavage activity by the RuvC domain. It was suggested that the Nuc domain of Cas12a plays a role as an additional endonuclease^33^. However, the domain was necessary only for positioning the scissile phosphates without direct cleavage activity^27^. A single active site in the Cas12a and Cas12b RuvC domains is involved in the cleavage of both target and nontarget strands^27,34^. The structure of the cleaved target sequence was found to have staggered 5‘-overhangs in Cas12a, Cas12b, Cas12e, and Cas14^28,29,32,35^. It is worthwhile to note that Cas14 exhibits collateral ssDNA degradation activity upon recognition of a target sequence^30^.

### Type VI CRISPR system

The type VI CRISPR-Cas system possesses unique RNase activity^14^. Cas13a (C2c2 / VI-A) grabs only crRNA that carries a 20-nt target-binding sequence. A pair of helical 1 domain or higher eukaryotes and prokaryotes nucleobinding (HEPN) domains replace the RuvC domain in other Cas proteins and are involved in RNA maturation and target RNA cleavage^36^. Type VI targets ssRNA and requires a protospacer flanking sequence (PFS) instead of the PAM required for dsDNA unwinding. The target cleavage rate is higher when PFS is not complementary to C in the 5 ‘repeat region of the crRNA for Cas13a, that is, non-G^37^. The HEPN domain activated by target ssRNA cleavage also exhibits collateral activity toward nontarget ssRNAs^38^. The transacting nontarget ssRNA cleavage activity has been employed for signal amplifications in molecular diagnostic systems^39^.

### Editing mode of the CRISPR system

**Fig. 3:**
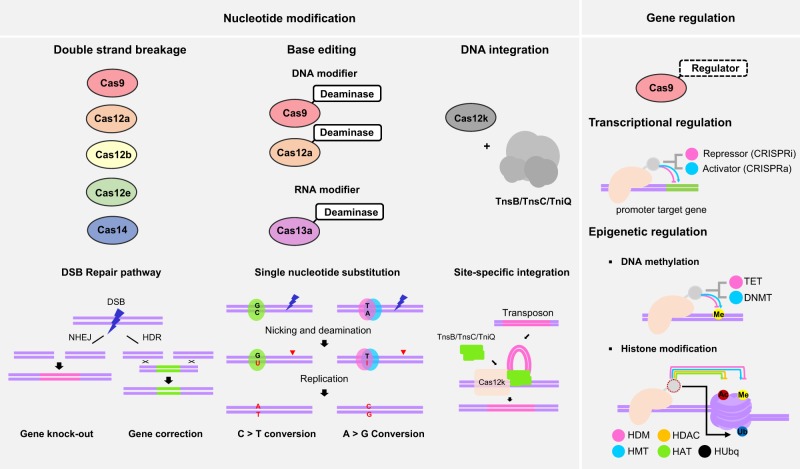
Modes of modifications in the nucleotide sequence and gene expression by the type II CRISPR system. Nucleotide sequence modifications are achieved by either a dsDNA cleavage-triggered repair process, base editing by Cas-deaminase fusion proteins, or DNA integrations by Cas12k-transposase complexes. The DSB repair process *per se* causes indel mutations through the NHEJ mechanism, and precise sequence modification can be achieved in the presence of template DNA via the HDR process. dCas or nCas can be fused with deaminases for hydrolyzing an amine group in cytosine and adenine. CBEs and ABEs were validated for DNA base editing, which induce C to T and A to G conversions, respectively. For RNA base editing, the adenosine deaminases acting on RNA (ADAR) enzyme was fused to dCas13 for reversible and temporal A to G conversion in RNA. Site-specific integration of transposable elements can be induced by transposase complexes under the guidance of catalytically dead Cas12k. The Cas 6/7/8 effector complex was suggested to replace the guide action of Cas12k in the presence of tnsA. Site-specific gene regulation is one of the main areas where genome editing tools can play a pivotal role. Transcriptional or epigenetic regulators can be linked to dCas directly by fusion or indirectly through viral RNA-protein interactions. Gene expression is either stimulated (CRISPRa) or inhibited (CRISPRi) depending on the functional nature of attached regulatory proteins. The site-specific gene regulatory effects can be made by either altered structural/network architecture in DNA or histone modifications

The eventual end for genome editing would be to achieve either qualitative or quantitative changes in gene expression. Indels of endogenous gene or integrations of exogenous DNA into a chromosome result in perturbations in gene expression either quantitatively or qualitatively. Qualitative changes can also be made by sequence substitutions via base editing or HDR-mediated gene corrections. The catalytically dead Cas (dCas) fused to a variety of functional proteins has been deployed for genetic and epigenetic regulations, thereby inducing quantitative changes in gene expression. The modes of how genome editing tools induce quantitative or qualitative changes in gene expression will be described in this section (Fig. 3). We do not provide detailed descriptions of biological processes underlying the modes but focus on the implications of each mode for a specific aim.

### DSB repair modes

The guide-RNA-dependent nuclease activity of the CRISPR-Cas system induces DSBs at the target site. DSBs are intracellularly recognized as severe damage and trigger DNA repair systems that are programmed. DSB repair can be achieved by either nonhomologous end joining (NHEJ) or homology direct repair (HDR) pathways. The former is a repair process that is driven without template DNA and thus often causes indel mutations. This process is used for gene ablations. In contrast, HDR is used to correct the target sequence to the intended sequence using donor DNAs with a certain length of homology arms^40^. HDR induces sequence substitutions or insertion of the designed nucleotide sequence into a targeted site. Alternatively, microhomology-mediated end joining (MMEJ), which replaces canonical NHEJ, uses a microhomology sequence of 5–25 nt to link the DSB ends and the gap between homologous sequences and leads to deletion of variable lengths^41^. Each repair process is differentially regulated depending on the stage of the cell cycle. HDR is limited to S/G2, while NHEJ is prevalent over the entire cell cycle period^42^. It was suggested that a high incidence of NHEJ can be used to insert DNA efficiently in a predesigned manner, wherein homology-independent targeted integration (HITI) was achieved with rates higher than that of HDR efficiency using a donor template without a homologous arm^43^. In addition, ssODN with homology arms of ~40 nt was shown to create higher HDR efficiency than dsDNA^44^. Relatively long DNA insertions (up to 1.5 kb) were achieved with ssDNA as a donor by basically using the HDR process^45^.

### Precise base editing with programmable deaminases

Precise gene correction is difficult to achieve with NHEJ because of its stochastic property. HDR-mediated correction is also unsatisfactory because the efficiency remains low. As an alternative tool, programmable deaminases, or base editors, were developed to address those limitations^46^. Base editors use a catalytically inactive Cas9 (dCas9 with D10A and H840A mutation) or nickase Cas9 (nCas9 with D10A mutation), which is fused to deaminase and hydrolyzes the amine group of C^47^ and A^48^. Cytidine base editors (CBEs) and adenine base editors (ABEs) were developed by two groups, and they enable ‘C to T’ and ‘A to G’ conversions, respectively, without dsDNA cleavage of DNA backbones. A third-generation base editor (BE3) is the mainly used form of CBEs and is a product of cytidine deaminase-nCas9-uracil DNA glycosylase inhibitor (UGI) combinations. dCas9 retains the ability to unwind target dsDNA, where APOBEC1 catalyzes deamination of Cs in the nontarget strand. The resultant U · G base pair is converted into a T · A base pair in the course of DNA replication. The U · G base pair frequently returns to the C · G pair after AP site generation by uracil N-glycosylase (UNG) and subsequent base excision repair. UGI prevents this process, and additional nicking of the target strand by nCas9 further increases the efficiency of BEs by facilitating mismatch repair^47^. *E. coli* tRNA adenosine deaminase enzyme (TadA) was deployed for ABEs to catalyze the first ‘A to inosine (I)’ conversion step^48^. The created I is recognized as G by DNA polymerase. The most widely used ABE 7.10 is a fusion form of heterodimeric TadA (wtTadA-mutantTadA) and nCas9. Each base editing tool offers different editing options according to the type of deaminase, nuclease, and gRNA. Depending on the Cas analogs or engineered forms, editing windows and PAM preference are varied^49^. Typically, BE3 and ABE7.10 show the highest editing efficiency in the window of protospacer position 4–8 and 4–7 (counting the PAM as positions 21–23)^47,48^. CBEs using LbCpf1 show editing window preference of positions 10–12^50^. The use of engineered APOBEC and nCas9 results in flexibility in the range of the editing window^49^. For example, APOBEC1 with W90Y, R126E, and R132E mutations allowed base editing in narrower windows of positions 5–6^51^. xCas9 and Cas9-NG can also expand the range of editable targets by lowering the restrictions on PAM^52,53^. Increased spacer length of the gRNA may result in a shift of the editing window^54^. In contrast to permanent genomic conversions by conventional base editors, the adenosine deaminases acting on RNA (ADAR) enzyme fused to dCas13 enables reversible and temporal control of ‘A to G’ RNA editing^55^.

### DNA integration using transposase

Targeted DNA integration is widely used in basic research and commercial applications because it not only guarantees safe harboring of exogenous DNA but also facilitates carrying out functional genetic studies without the interference of internal genetic changes. HDR-mediated gene insertion has been the first-line approach, where programmable nucleases trigger DSB at a targeted site, and then ssDNA or dsDNA donors are integrated through a double crossover event. However, we still cannot expect a high level of integration efficiency despite the introduction of several technical approaches^56–58^. It has recently been suggested that HITI improves the efficiency of integrating larger DNA fragments, but the integration through this mechanism is bidirectional^43^.

Recently, two DNA integration methods using transposons were proposed as an alternative tool to address these problems. These methods use a catalytically inactive Cas effector protein. Tn7-like transposons are known to be associated with subtypes such as type I-B, I-F, and V-K (V-U5) Cas proteins^59^. CRISPR-associated transposase (CAST) relies on Cas12k for gene targeting, which then interacts with tnsB/C and tniQ^59^. Multiple complexes composed of Cas 6,7,8 effectors, tnsA/B/C and TniQ, were also validated for this purpose^60^. These two techniques achieved targeted integration 49–66 bp downstream of G- and T-rich PAM and 46-55 bp downstream of C-rich PAM, respectively. The left end (LE) and the right end (RE) of ~200 bp are required at both ends of the transposons for integration of up to ~10 kb. This transposase-dependent DNA integration does not provoke DSBs in the genome, which may guarantee safer and more specific DNA integration. Moreover, the integration efficiency was sufficient to replace HDR-mediated integration. However, these methods were not validated in eukaryotic cells, and validation efforts in such settings should be carried out in the future. Moreover, the issue of Cas12k-independent off-target integrations should also be addressed to ensure bona fide specific, targeted DNA integrations.

### Gene regulation using the CRISPR-dCas system

Unlike the previously described editing modes that involve DNA sequence modifications, this strategy relies on dCas deprived of catalytic activity but retains a gRNA-assisted gene targeting property. Instead of repair mechanisms following DSBs, this approach depends on the functional role of regulatory proteins fused to dCas. Such created dCas-regulatory fusion proteins act as site-specific regulators of gene expression. Depending on the function of fused regulatory proteins, gene expression can be either activated (CRISPRa) or interfered (CRISPRi). For CRISPRa, transcription activators such as VP64, P65, and Rta have been deployed. They can be either directly fused to dCas9 or recruited to a target site via interactions between viral RNA-viral RNA recognizing protein^61^. In the latter, the regulatory proteins are fused to the viral RNA recognizing protein, which, in turn, binds to gRNA decorated with viral RNA modules. This strategy offers multiplexed and reinforced gene activations^61,62^. In addition, SunTag, a repeating peptide array capable of recruiting antibody fused proteins, can be coupled to dCas to recruit multiple activators for maximized activation^63^. In contrast, the inhibitory effects of CRISPRi basically originate from interference with RNA polymerase activity by locally resident dCas9^61^. The extent of interference can vary depending on the selection of target sites and directions among regulatory regions and was further increased by involving a transcription repressor, such as KRAB^64^.

Alternatively, site-specific epigenetic regulations have been pursued to achieve altered expression of target genes. These include target-specific DNA demethylation using Tet1^65^ enzyme and reversal DNA methylation using DNMT^66^, particularly near CpG islands in the promoter regions. In addition, histone acetylation inducers such as p300 and LSD1 weaken the interaction between histone and DNA binding sites to facilitate access to transcription factors^67,68^. The methylation of histone protein may have different effects depending on the methylated amino acid residues: K4 methylation of H3 upregulates gene expression, while K27 methylation is inhibitory^69^. The ubiquitination of histone can be aimed at promoting gene expression. These approaches are used not only to regulate gene expression but also to elucidate the causal relationship between the various epigenetic marks and the phenomena that occur as a result of regulating these genes.

### Present shortcomings and technical developments

CRISPR technology ‘democratizes’ the genome editing field on the basis of its relatively high efficiency, easy accessibility to users, simplicity of use, compatibility with genetic screening, etc. These technical merits have allowed the adoption of technology in various fields, including basic science and commercial applications. Despite the outstanding performances of CRISPR, there are several shortcomings that need to be further addressed for full-fledged use CRISPR. In fact, considerable efforts to refine CRISPR technology have been made to surmount these technical hurdles. In this section, the shortcomings will be described with related developmental achievements and directions for further refinements.

### Insufficient indel and HDR efficiency

One main feature that allowed the rapid acceptance of CRISPR technology is its high DNA modification efficiency. CRISPR has shown unsurpassed indel efficiency in a variety of cells compared to the other programmable nucleases. Several studies have even presented indel values almost reaching 100%^70^. Nonetheless, it is clear that there are target sites that show exceptionally low indel efficiency. Moreover, a clear interpretation of such ‘target to target’ variations is lacking. To address this issue, efforts to increase indel efficiency have been made by engineering either Cas^25^ or gRNA^71^. Alternatively, artificial intelligence (AI)-based deep learning algorithms have been adopted to predict target regions with potentially high indel efficiency (http://deepcrispr.info)^72^. Deeper knowledge regarding the repair mechanism and chromatin structures would provide opportunities to achieve steady and sufficient DNA modification efficiency.

Even more importantly, there remain technical limitations for gene corrections using base editors or HDR. Although base editing shows relatively high conversion efficiency, only C to T and A to G conversions are currently feasible by CBEs and ABEs, respectively. The development of substitutive tools, for instance, for ‘C to A’ or ‘T to A’ conversions, would provide further opportunities for DNA modifications at a resolution of a single nucleotide. Furthermore, HDR efficiency remains low, although there have been increases in the efficiency by supplementing chemical reagents, such as SCR7^73^, RS-1^74^, KU0060648, and NU7441^75^. The use of a donor template in the form of ssDNA or gRNA-donor DNA fusion also led to increased HDR efficiency^76,77^. Further refinement of related methods, such as HITI and transposase-based integration, would lead to a breakthrough in this realm.

### Off-target issue

The off-target issue has become a mainstay in efforts to improve the CRISPR system, particularly for therapeutic uses. Off-target genome editing refers to DNA modifications at unintended and nonspecific sites and can occur by misguides by gRNA or in a gRNA-independent manner^78,79^. Efforts to address off-target editing have been made in largely two directions: developing an off-target detection method and engineering the CRISPR system for high-fidelity editing.

The assessment of off-targets can be conducted by either a biased or a genome-wide unbiased analysis. For a biased off-target analysis, several bioinformatics tools, such as Cas-OFFinder and CCTop (https://crispr.cos.uni-heidelberg.de), were developed to predict potential off-targets with similar sequences and PAM compatibility. An NGS-based sequencing of PCR-amplified potential off-target regions enables deep sequencing, which is otherwise unattainable by unbiased analyses. In contrast, unbiased off-target analyses have been developed to probe for unintended off-target mutations throughout genomes and thus help estimate the overall level of specificity genome-wide. To date, various methods have been developed, including SELEX, IDLV capture, Guide-seq, HTGTS, BLESS, Digenome-seq^80,81^, and DISCOVER^82^. Some of them are based on in vitro treatments of genomic DNA, while others enable in situ or in vivo assessments^81^. In addition, some of them can be applied to off-targets for DSB, whereas others are applied to unintended base editing^83,84^. Researchers may have to adopt a suitable analytic tool for their purpose because each tool has respective *pros and cons*.

Significant improvements to decrease off-target activity of CRISPR tools have been made. First, Cas proteins that show improvements in on-target specificity were engineered, which include eSpCas9^85^, HF-Cas9^86^, HypaCas9^87^, and Sniper Cas9^88^. eSpCas9, HiFiCas9, and HypaCas9 were developed by rational design for structural modifications to increase specificity, whereas Sniper Cas9 was screened from a library of SpCas9 mutants that showed increased specificity. Most of the engineered Cas proteins displayed remarkably reduced off-target levels while retaining on-target activity. Increased specificity has also been achieved by gRNA engineering (for in-depth information, please refer to a review article^71^).

Despite these endeavors, more work is necessary to possess sufficiently safe genome editing tools. In particular, it was recently reported that CBE has random and unpredictable off-target effects in a gRNA-independent manner^78,79^. Moreover, it is involved in the modifications of cellular RNA^89^. These nonspecific base editing properties of CBE and ABE were improved in terms of off-target activity as well as on-target editing efficiency through the replacement of the deaminase enzyme with human APOBEC3A or selective mutations for APOBEC and tadA-tadA* proteins^90^. As mentioned above, the issue of random insertion in HDR or transposase-mediated integration remains an area for further improvement^59^.

### Large size in gene delivery

The first step for genome editing is to prepare CRISPR components comprising Cas or Cas derivatives, gRNA, and, when necessary, additional DNAs, etc. These components will then be delivered inside cells. Vehicles for delivery can be largely divided into viral and nonviral vectors. Nonviral delivery includes microinjection, electroporation, or the use of chemicals^91^. Depending on the delivery system, genome editing components can take various forms among DNA, RNA, or the ribonucleoprotein complex (RNP). Diverse kits and instrumentations have been developed for each delivery option. However, in vivo gene therapy usually, if not always, relies on the viral delivery system, and adeno-associated viruses (AAVs) have been suggested to be a preferred viral vector. There is, however, a limitation in the loaded gene size (~4.5 kb) for efficient delivery. The problem is that most of the validated Cas proteins are too heavy. For instance, SpCas9 has 1368 amino acids, and it is difficult to pack both Cas9- and gRNA-coding DNA into a single AAV particle. This challenge has been addressed in two ways. One was to search for lightweight Cas orthologs from archaea and bacteria, which include NmCas9 (1082 aa), SaCas9 (1053 aa), CjCas9 (984 aa)^62^ and ScCas9^92^. They were loaded into several AAV serotypes and effectively delivered in vivo^93–95^. Cas14 can be a player in such lightweight members because it was recently reported to retain dsDNA cleavage activity^32^. Alternatively, heavy SpCas9 was split into N-Cas9 (2–573 aa) and C-Cas9 (574–1368 aa), each of which was loaded into separate AAVs^22^. The reconstituted Cas9 was successfully delivered in vivo as well as into cultured cells.

Genome editing tool sets include not only dsDNA cutters but also base editors and dCas-based gene regulators used for CRISPRi and CRISPRa. The latter means that further increases in the full gene size are inevitable. Although these Cas-regulator fusion modules have not been tested in an AAV viral vector system, sophisticated technical developments would open the phase where we could deliver various genome editing tools using viral vector systems without concern for size.

### Target restriction and PAM variability

A DSB is preceded by the recognition of a PAM sequence by Cas proteins^53,62^, and each Cas protein has its own preferred PAM sequence. As a rule of thumb, type II CRISPR-Cas usually recognizes 3’ G-rich sequences, while type V recognizes 5’ T-rich sequences. One may choose a suitable tool in a context-dependent manner from a genome editing toolbox because each Cas ortholog has slightly different PAM specificity. Nonetheless, there are more than a few cases where intended DNA modifications are difficult to achieve due to an unavailable PAM. These cases are frequently encountered when using base editors with fixed editing windows. PAM variants of Cas help expand the range of editable targets by decreasing PAM restrictions. Structurally informed engineering yielded a shift in the PAM preference of SpCas9^96^. Cas-NG and xCas are engineered Cas9 variants that basically recognize an NG PAM sequence^52,53^. Cas12a-derived PAM variants were also developed for such purposes with RR and RVR variants recognizing TYCV and TYTV PAMs, respectively^97^. The engineered PAM variants are confined to SpCas9 and Cas12a. If extended to other orthologs, such as CjCas9 or Cas14, such efforts will provide flexibility in the target selection.

### Immune response

In general, CRISPR tools work by incorporating prokaryote-derived biomolecules into eukaryotic cells and bodies. This should raise concern that foreign substances may provoke cellular toxicity and immune responses. In fact, researchers from Stanford University analyzed blood samples and found that 79% of the participants had antibodies against SaCas9, and 65% were positive for antibodies against SpCas9^98^. Two Cas orthologs were the most extensively studied proteins for gene therapy. SaCas9 and SpCas9 were derived from *S. aureus* and *S. pyogenes*, which are prevalent in human environments and thus prone to contact with the human immune system. The journal *Nature* directly noted this issue, stating that “researchers hoping to use a gene-editing technique to treat disease may have to seek alternative enzymes”^98^. A more careful approach may be needed to use Cas proteins, particularly for in vivo gene therapy. Cas from organisms less contagious to humans should be further sought and tested in terms of immunogenicity. Notably, the 5’-terminal phosphate group in gRNA triggers an innate immune response in human cells^99^. The modification of the phosphate into a 5’-hydroxyl group was suggested as a means of blocking undesirable biological responses.

## Conclusion

We have witnessed dramatic progress in genetics and molecular biology during the last century. Researchers have unveiled the structure of DNA, how DNA codes for protein synthesis, and how DNA sequences can be delineated using Sanger sequencing analysis and next-generation sequencing methods. The time has come when one-day diagnosis can be made, where whole-genome sequencing and interpretations on the genotype-disease associations are conducted in a single day. We would like to stress that these technologies are centered on “DNA reading”. Now we are on the move toward the “DNA writing” era with the aid of genome editing technology, and CRISPR technology is indeed opening up this era.

The ‘DNA writing’ nature of CRISPR technology has allowed its use as a DNA rewriting tool. For basic study, cells or zygotes have been objects for DNA writing to generate model cell lines and animals. The concept of ‘gene drive’ is becoming realized by CRISPR-Cas9, where disease-carrying mosquitoes were targeted for DNA rewriting^100^. When certain genes in plants are rewritten, disease- or abiotic stress-resistant varieties can be developed^101^. The specific targeting nature of Cas protein, combined with collateral nuclease activity, was also utilized as a molecular diagnostic tool in a highly sensitive manner^102^. Above all, gene therapy is the mainstay for this DNA rewriting endeavor. During the course of long-term evolution, nature has accumulated various genetic mutations in living organisms. These mutations have been the driving force for natural selection and genetic diversity. However, there are patients who suffer from various genetic disorders. CRISPR technology can offer therapeutic opportunities for treating such rare genetic diseases, in which *ca*. 3,700 different mutations are involved. With a few clinical trials taking place at present, we will soon witness a plethora of gene therapy technologies conducted worldwide using CRISPR tools.

It is worthwhile to stress that each application requires different levels of functionality of the CRISPR system. For example, size and immune response may not be serious matters in plant biotechnology. Rather, HDR efficiency or low off-target activity may be critical requirements to obtain desirable traits. On the other hand, these issues should be carefully controlled for applications to gene therapy. As described earlier, CRISPR still has several technical challenges. It may take a long time until we develop a ‘super’ CRISPR tool that shows excellent efficiency while still having acceptable levels of specificity and safety. Thus, it is desirable to focus on traits that need to be addressed for specific applications. If these technical shortcomings are fully addressed on an application basis, DNA writing applications will flourish with a toolbox of elaborate CRISPR technologies.
